# Pathological Mechanisms and Preventive Strategies of Oxaliplatin-Induced Peripheral Neuropathy

**DOI:** 10.3389/fpain.2021.804260

**Published:** 2021-12-08

**Authors:** Nobuaki Egashira

**Affiliations:** Department of Pharmacy, Kyushu University Hospital, Fukuoka, Japan

**Keywords:** cancer, chemotherapy, oxaliplatin-induced peripheral neuropathy, peripheral neuropathy, neuropathic pain, oxaliplatin, side effects, animal models

## Abstract

Oxaliplatin, which is widely used in treating cancers such as colorectal cancer, frequently causes peripheral neuropathy. It not only significantly reduces the patient's quality of life due to physical distress but may also result in a change or discontinuation of cancer treatment. Oxaliplatin-induced peripheral neuropathy (OIPN) is classified as acute or chronic depending on the onset time of side effects; however, the prevention and treatment of OIPN has not been established. As these peripheral neuropathies are side effects that occur due to treatment, the administration of effective prophylaxis can effectively prevent their onset. Although transient relief of symptoms such as pain and numbness enable the continuation of cancer treatment, it may result in the worsening of peripheral neuropathy. Thus, understanding the pathological mechanisms of OIPN and finding better preventative measures are important. This review focuses on animal models to address these issues, clarifies the pathological mechanisms of OIPN, and summarizes various approaches to solving OIPN, including targets for preventing OIPN.

## Introduction

Anticancer drugs, such as vinca alkaloids (e.g., vincristine, vinorelbine, and vinblastine), taxanes (e.g., paclitaxel and docetaxel), platinum derivatives (oxaliplatin and cisplatin), and bortezomib are commonly used in cancer chemotherapy; however, they typically induce peripheral neuropathy, characterized by numbness and pain in the limbs. Chemotherapy-induced peripheral neuropathy (CIPN) is a serious, dose-limiting adverse effect of cancer chemotherapy. These neuropathies not only significantly lower the patient's quality of life, but also force them to change or discontinue anticancer drug treatment, which poses a major clinical problem (1). However, the mechanisms of CIPN have not been fully clarified, and according to the American Society of Clinical Oncology clinical practice guidelines, there is no established treatment strategy for preventing or treating CIPN (2, 3). Therefore, elucidating the mechanisms of CIPN through basic research and establishing effective preventive measures and treatment methods are important. In addition, drug repositioning is currently being practiced because it can be applied instantly in clinical settings (4).

This review focuses on oxaliplatin-induced peripheral neuropathy (OIPN), which is of particular concern in clinical practice. We then summarize the pathological mechanisms of OIPN in animal models, various approaches to solving OIPN, and potential targets for preventing OIPN to provide direction for future strategies.

## Characteristics of OIPN

### Features

Oxaliplatin is an important drug in the treatment of cancers such as colorectal cancer. However, it frequently induces peripheral neuropathies, which are classified as acute or chronic depending on whether the effects appear immediately/within a few days after drug administration or at a later stage, respectively (1, 5). The major symptoms of acute peripheral neuropathy are paresthesia around the limbs, mouth, pharynx, and larynx and slight motor dysfunction. These symptoms are more likely to occur or worsen due to low temperature stimulation. Such acute peripheral neuropathy is characteristic of the administration of oxaliplatin but not other platinum derivatives. In contrast, chronic peripheral neuropathy presents with persistent numbness, paresthesias, and pain in the limbs, and in advanced cases, it becomes difficult to write letters, hold chopsticks, press buttons, or even walk. Importantly, the motor dysfunction interferes with daily life. This is also seen in peripheral neuropathy caused by other platinum drugs, such as cisplatin.

### Approaches

Preventing OIPN not only improves the patient's quality of life, but also increases the likelihood of continued treatment. Taking measures against peripheral neuropathy is important because the completion of postoperative adjuvant therapy decreases recurrence rate. As a treatment for OIPN, it is common to initially observe sensory abnormalities such as cold sensitivity and numbness of the hands carefully, and if it is difficult to continue treatment, appropriate measures such as dose reduction or drug suspension are taken. Drugs, such as pregabalin and duloxetine, other serotonin-noradrenaline reuptake inhibitors, and tricyclic antidepressants may be used; however, their effectiveness is insufficient. Thus, there is no established preventive/therapeutic method for OIPN. Therefore, it is desirable to elucidate the mechanisms involved in OIPN occurrence and establish effective preventive measures and treatment methods.

## Mechanisms of OIPN in Animal Models

### Mechanisms of Acute Peripheral Neuropathy

Oxaliplatin is metabolized *in vivo*, and oxalate is separated from dichloro (1,2-diaminocyclohexane) platinum [Pt (dach) Cl_2_]. In animal experiments, administration of oxalate (sodium oxalate) causes acute cold allodynia, similar to the administration of oxaliplatin (6). However, administration of oxalate did not induce mechanical allodynia that occurred at a later stage with oxaliplatin administration. Thus, oxalate is involved in cold allodynia, an acute peripheral neuropathy, which can also be caused by oxaliplatin. Oxalate, a chelating agent for intracellular Ca^2+^, acts on the membrane potential of neurons and alters activities of both voltage-gated ion channels and transient receptor potential (TRP) channels (7). Oxaliplatin-induced cold allodynia is associated with increased expression of TRP melastatin 8 *via* the Na^+^ channel/Ca^2+^ channel/nuclear factor of activated T-cell pathway (8). Oxalate acts on Na^+^ channels to increase action potentials and induce Ca^2+^ influx into the dorsal root ganglion (DRG), thereby increasing the translocation of nuclear factor of activated T-cell into the nucleus and expression of TRP melastatin 8. Nifedipine (an L-type Ca^2+^ channel blocker), diltiazem (an L/T-type Ca^2+^ channel blocker), and mexiletine (a Na^+^ channel blocker) suppressed the expression of TRP melastatin 8 in the DRG and prevented cold allodynia in rats. Furthermore, a survey of the medical records of 69 male patients who received the oxaliplatin regimen for colorectal cancer, which is a combination therapy of fluorouracil, leucovorin, and oxaliplatin, confirmed that the incidence of acute peripheral neuropathy was significantly lower in patients taking Ca^2+^ channel blockers than in patients not taking Ca^2+^ channel blockers (9). Oxaliplatin-induced cold allodynia is also associated with cold sensitivity mediated by TRP ankyrin 1 (TRPA1). Oxaliplatin enhances the sensitivity of TRPA1 by inhibiting prolyl hydroxylase, an enzyme that hydroxylates proline in the N-terminal ankyrin repeat of TRPA1 (10). Oxaliplatin also reduces intracellular pH in the DRG neurons and sensitizes TRPA1 (11, 12). In addition, tandem of pore domains in a weak inward rectifying potassium channel-related potassium channel 2 and hyperpolarization-activated cyclic nucleotide-gated 1 channels are involved in oxaliplatin-induced cold allodynia (13, 14).

### Mechanisms of Chronic Peripheral Neuropathy

The accumulation of platinum compounds in nerve cells damages ganglion cells and nerve fibers and causes chronic peripheral neuropathy (15). In animal experiments, the administration of Pt (dach) Cl_2_ alone does not cause cold allodynia, but mechanical allodynia may later develop (6). This indicates that platinum-containing compounds are involved in chronic OIPN. Oxaliplatin caused cold allodynia without axon degeneration within a few days of administration, whereas repeated injection of oxaliplatin caused mechanical allodynia and axon degeneration in the sciatic nerves of rats a few weeks after the start of administration (16), suggesting that axon degeneration in rat sciatic nerve is associated with mechanical allodynia. Furthermore, in the spinal cord of rats, in which oxaliplatin caused mechanical allodynia, increased expression levels of the *N*-methyl-D-aspartate (NMDA) receptor subtype 2B (NR2B) and its downstream targets, nitric oxide synthase (NOS) activity, and phosphorylation of Ca^2+^/calmodulin-dependent protein kinase II (CaMKII) were observed (17, 18). In addition, NMDA receptor antagonist (MK-801), NR2B inhibitor (Ro 25-6981), NOS inhibitor (L-NAME), neuronal NOS inhibitor (7-nitroindazole), and CaMKII inhibitor (KN-93) suppressed oxaliplatin-induced mechanical allodynia. Furthermore, memantine, an NMDA receptor antagonist, and ifenprodil and trifloperazine, which have NR2B and calmodulin inhibitory effects, respectively, suppressed CaMKII activity and temporarily improved mechanical allodynia. These findings suggest that the NR2B-mediated activation of NOS and CaMKII is involved in oxaliplatin-induced mechanical allodynia. Moreover, a significant increase in extracellular glutamate levels and a decrease in glutamate transporter 1 expression were observed in the spinal cord of rats during the development of mechanical allodynia induced by oxaliplatin (19). In contrast, activation of spinal astrocytes is associated with induction, not maintenance of oxaliplatin-induced mechanical allodynia (20).

Oxaliplatin uptake into the DRG causes excessive oxidative stress in the sciatic nerve of rats, followed by nerve damage, such as axonal degeneration. Hypomyelination is observed in the sciatic nerve of rats, and this decrease in the myelin sheath may be associated with changes in β-secretase 1 and neuregulin 1 (21, 22).

In one study, while transporting platinum to the DRG, the overexpression of organic cation transporter (OCT) 2 significantly increased oxaliplatin cell uptake and DNA platination. OCT2 is predominantly expressed in satellite glial cells, and genetic and pharmacological knockout of OCT2 protected against oxaliplatin-induced neurotoxicity in mice (23, 24). In addition, the drug transporters, OCT novel (OCTN) 1 and multidrug and toxic extrusion 1 (MATE1), are involved in platinum accumulation in the DRG and OIPN (25, 26).

Oxaliplatin also causes morphological changes in the peripheral nerve terminals in the skin and mitochondrial dysfunction in peripheral nerve axons (27, 28). Acetyl-l-carnitine and olesoxime, drugs that protect mitochondria, suppress the development of oxaliplatin-induced neuropathy in rats (27, 28). Furthermore, nuclear factor-erythroid 2-related factor 2, a transcription factor which plays an important role in the maintenance of mitochondrial homeostasis, inhibited OIPN *via* protection of mitochondrial function in mice (29). Thus, mitochondrial dysfunction is a key contributor to OIPN.

## Best Approach to OIPN

### What Is the Best Prevention Approach?

Goshajinkigan (GJG) is a Kampo medicine prescribed for treating lower limb pain, back pain, and numbness and is typically used for CIPN treatment in Japan. In an animal model of OIPN, prophylactic injection of GJG prevented cold allodynia but did not prevent mechanical allodynia and axonal degeneration in rat sciatic nerves (30). Furthermore, a single administration of GJG transiently reduced both cold- and mechanical allodynia after the development of neuropathy. Thus, GJG may relieve OIPN. In a placebo-controlled, double-blind, randomized phase III study, GJG did not prevent OIPN, but the time to reach grade 2 or greater sensory neuropathy was shortened in the GJG-treated group (31). Notably, the dose intensity and treatment cycle were higher in the GJG-treated group than in the GJG non-treated group. These results suggest that increased oxaliplatin doses are administered to patients because GJG transiently relieves peripheral neuropathy. However, GJG does not have a protective effect on nerves, and consequently, its analgesic effect may result in the exacerbation of OIPN, including motor dysfunction. Therefore, protecting or repairing nerve damage rather than using analgesic or palliative treatment for OIPN is crucial.

In addition, CIPN is the only neuropathy that can be prevented from developing because it is caused by cancer chemotherapy. Therefore, it is appropriate to prioritize the prevention of onset rather than analgesia or palliative care as a treatment strategy for CIPN. One approach to prevent OIPN is to reassess oxaliplatin-based chemotherapy regimens. In oxaliplatin-based chemotherapy, the incidence of long-lasting peripheral sensory neuropathy was significantly lower in patients receiving treatment for 3 months than in those receiving treatment for 6 months, and significantly lower in patients receiving capecitabine plus oxaliplatin therapy than in patients receiving fluorouracil, leucovorin, and oxaliplatin combination therapy (32). Therefore, a 3-month course of capecitabine plus oxaliplatin may be the most appropriate treatment option, particularly for low-risk patients. The second approach to prevent OIPN is to administer prophylactic drugs to prevent the development of neuropathy. These drugs need to not only suppress pain sensations but also nerve damage. In addition, since these drugs are administered prophylactically, it is desirable to use oral drugs which have fewer side effects than injectable drugs. It is also essential that they do not affect the antitumor effects of anticancer drugs. However, treatments that repair damaged tissues are also an important strategy for peripheral neuropathy.

### What Is the Best Research Approach?

Paclitaxel increases substance P release in cultured adult rat DRG cells (33). The antiallergic drug, pemirolast, suppresses this release and temporarily suppresses paclitaxel-induced peripheral neuropathy in rats. In contrast, pemirolast does not improve OIPN in rats. Notably, oxaliplatin did not release substance P in cultured DRG cells (33). These findings indicate that substance P is less involved in OIPN. Thus, despite the development of similar neuropathic symptoms, each chemotherapeutic drug causes CIPN through a different mechanism. Therefore, understanding drug-specific mechanisms and developing drug-specific strategies for CIPN are necessary. In addition, clarifying the detailed mechanisms of CIPN and identifying candidate drugs for CIPN based on these mechanisms are important.

Many clinical trials are currently underway to evaluate the effects of drugs against OIPN (34). Drug repositioning studies apply approved drugs used in clinical practice for new uses to reduce safety concerns and the time and cost of drug development (35). Therefore, drug repositioning is an effective approach for the “development” of drugs for OIPN.

It is necessary to explore potential OIPN drugs for protective or reparative effects on nerve damage using nerve cells (e.g., DRG cells and PC12 cells) and animal nerve tissues (e.g., the sciatic nerve and DRG) (16). Furthermore, evaluating pain sensations in animals and the effects of the proposed therapeutic agents on OIPN and on the antitumor action of oxaliplatin (30) is important.

### Targets for the Prevention of OIPN

Recently, a number of studies have reported on the prevention of OIPN in animal models. Table 1 summarizes the candidate drugs for the prevention of OIPN. The main targets for prevention can be divided into “neuroprotection” and “drug transportation”.

**Table 1:** Candidate drugs for the prevention of oxaliplatin-induced peripheral neuropathy (OIPN).

**Targets**	**Drugs**	**Dose**	**Animal model**	**Symptoms targeted by the drug**	**References**
Neuroprotection	Dimethyl fumarate	200 mg/kg	Rats	Mechanical allodynia and axonal degeneration of the sciatic nerve	(36)
	Donepezil	1 mg/kg	Rats	Mechanical allodynia and axonal degeneration of the sciatic nerve	(37)
	Alogliptin	10 mg/kg	Rats	Mechanical allodynia and axonal degeneration of the sciatic nerve	(38)
	Neurotropin (a non-protein extract derived from the inflamed skin of rabbits inoculated with vaccinia virus)	200 NU/kg	Rats	Mechanical allodynia and axonal degeneration of the sciatic nerve	(16)
	Ibudilast	7.5 mg/kg	Rats	Mechanical allodynia and axonal degeneration of the sciatic nerve	(39)
	Tadalafil	10 mg/kg	Mice	Mechanical, cold, and electrical current hypersensitivities; thermal hypoesthesia; and axonal degeneration of the sciatic nerve	(40)
	Benztropine	10 mg/kg	Mice	Mechanical and cold hypoesthesia, decreased nerve SCV, and demyelination in the sciatic nerve	(41)
	Niclosamide	10 mg/kg	Mice	Mechanical hypoesthesia and cold hyperalgesia, IENF density reduction, and demyelination in the sciatic nerve	(42)
	A low-molecular-weight MnSOD mimic molecule	10 mg/kg	Mice	Mechanical hypoesthesia and demyelination in the sciatic nerve	(43)
	Cystine/theanine	280 mg/kg	Rats	Mechanical allodynia and axonal degeneration of the sciatic nerve	(44)
	Fulvestrant	10 mg/kg	Rats	Mechanical allodynia and axonal degeneration of the sciatic nerve	(45)
	Tanshinone IIA	25 mg/kg	Rats	Mechanical allodynia and sciatic nerve dysfunction	(46)
	Topiramate	100 mg/kg	Rats	Mechanical allodynia, decreased nerve SCV, caudal nerve fibers density reduction, and IENF density reduction	(47)
Nerve repair	Exenatide	0.1 mg/kg	Rats	Mechanical allodynia and axonal degeneration of the sciatic nerve	(48)
Drug transporters	Cimetidine	30 mg/kg	Mice	Mechanical allodynia and cold hypersensitivity	(23)
	Dasatinib	15 mg/kg	Mice	Mechanical allodynia and cold hypersensitivity	(49)
	Ergothioneine	15 mg/kg	Rats	Mechanical allodynia	(25)
Others	RJW-58 (a cathepsin S inhibitor)	25 mg/kg	Mice	Mechanical allodynia, demyelination in the sciatic nerve, and IENF density reduction	(50)
	Small extracellular vesicles	3 ×10^11^ particles/injection	Mice	Mechanical allodynia, cold hyperalgesia, decreased nerve SCV, IENF reduction, and demyelination in the sciatic nerve	(51)

*IENF, Intraepidermal nerve fiber; MnSOD, manganese superoxide dismutase; NU, neurotropin unit; SCV, sensory conduction velocity*.

#### Neuroprotection

OIPN involves nerve damage, such as axonal degeneration of the sciatic nerve. Therefore, achieving neuroprotective effects is a target for preventing OIPN. Dimethyl fumarate, an oral drug for multiple sclerosis, activates the nuclear factor-erythroid 2-related factor 2 pathway and prevents oxaliplatin-induced mechanical allodynia and axonal degeneration of the sciatic nerves in rats (36, 52). Donepezil, an oral drug used for treating Alzheimer's disease; alogliptin, a dipeptidyl peptidase-4 inhibitor and oral antidiabetic drug; and neurotropin, a non-protein extract which is used to treat various chronic pain, prevent oxaliplatin-induced mechanical allodynia and axonal degeneration of the sciatic nerve in rats (16, 37, 38). Neurite outgrowth as an indicator of axonal degeneration was analyzed using a cultured cell model (16, 37). All these drugs improve the oxaliplatin-induced inhibition of neurite outgrowth in cultured pheochromocytoma PC12 cells or primary cultured rat DRG neurons (37, 38, 52). Ibudilast, a non-selective phosphodiesterase inhibitor used to treat bronchial asthma and dizziness after stroke, was recently reported to elongate the neurites in PC12 cells and prevent oxaliplatin-induced mechanical allodynia and axonal degeneration of the sciatic nerves in rats (39). Similarly, tadalafil, a phosphodiesterase 5 inhibitor, suppresses oxaliplatin-induced mechanical allodynia and axonal degeneration in mice (40). Benztropine, an inhibitor of acetylcholine muscarinic M1 and M3 receptors, niclosamide (an antihelminthic drug), a low-molecular-weight manganese superoxide dismutase (MnSOD) mimic molecule, and a new super-oxide dismutase modulator, prevented acute and chronic OIPN and demyelination in the sciatic nerves of mice (41–43). Moreover, cystine/theanine (a supplement) and fulvestrant, a drug approved for treating breast cancer in postmenopausal women, could prevent both mechanical allodynia and axonal degeneration induced by oxaliplatin in rats (44, 45). In addition, tanshinone IIA, a compound extracted from the medicinal herb *Salvia miltiorrhiza*, and topiramate, an antiepileptic drug, prevented oxaliplatin-induced neuropathic pain and axonal damage of sciatic nerves in rats (46, 47). In contrast, exenatide, a glucagon-like peptide-1 agonist and antidiabetic drug, facilitated recovery from oxaliplatin-induced mechanical allodynia by repairing axonal degeneration in rats (48).

#### Drug Transporters

Platinum accumulation in nervous tissues is the major mechanism responsible for OIPN. Therefore, transporters related to platinum transportation should be targeted. OCT2 is expressed in DRG cells and is involved in the cellular uptake of oxaliplatin (23). Genetic knockout of *OCT1/2* prevented cold- and mechanical allodynia, and cimetidine, an inhibitor of OCT2, protected against mechanical allodynia in wild-type mice treated with oxaliplatin. Moreover, dasatinib, a tyrosine kinase inhibitor and potent inhibitor of OCT2, inhibited platinum uptake in the DRG and mitigated mechanical allodynia and cold hypersensitivity in wild-type mice treated with oxaliplatin (49). OCTN1/2 and MATE1 are also expressed in the DRG, and these overexpressing cells show intracellular accumulation of oxaliplatin in human embryonic kidney 293 cells (26). The knockdown of OCTN1, but not OCTN2, in DRG decreased platinum accumulation in the DRG and weakly suppressed mechanical allodynia in rats treated with oxaliplatin. Conversely, the knockdown of MATE1 increased platinum accumulation in the DRG and resulted in more severe mechanical allodynia in rats. Moreover, ergothioneine, an OCTN1 substrate/inhibitor, decreased both oxaliplatin accumulation in the DRG and the development of mechanical allodynia in rats (25). However, L-carnitine, an OCTN2 substrate/inhibitor, did not provide the same results. These findings indicate that OCT2, OCTN1, and MATE1 could be targets for preventing OIPN.

#### Other Prevention Targets

Cathepsin S (lysosomal cysteine protease) and exosomes, the major constituents of small extracellular vesicles, have recently become targets for OIPN. Cathepsin S is essential for maintaining neuropathic pain through cleavage of the transmembrane chemokine, fractalkine (53). Exosomes play critical roles in intercellular communication (54). RJW-58, a cathepsin S inhibitor, and small extracellular vesicles derived from cerebral endothelial cells prevented mechanical allodynia and damage to the sciatic nerves in mice treated with oxaliplatin (50, 51).

## Discussion

Although various preventive and therapeutic agents are being tested recently for peripheral neuropathy caused by oxaliplatin, effective preventive and therapeutic methods have not been established. Various attempts have been made in the field, including the search for drugs that prevent the onset of peripheral neuropathy and drugs that alleviate the symptoms caused by peripheral neuropathy. However, relieving pain may lead to overlooking the exacerbation of peripheral neuropathy, does not protect nerve damage, and is not a radical cure. Therefore, OIPN can be better managed by drugs that prevent nerve damage rather than simply relieve pain. Hence, drugs that protect nerves or act on drug transporters to suppress the accumulation of platinum are targeted. Recent studies have also targeted cathepsin S and exosomes.

OIPN presents itself as either acute neuropathy or chronic neuropathy, and these neuropathies are not successive; thus, chronic neuropathy can occur even if acute neuropathy does not occur. Moreover, if acute neuropathy can be suppressed, chronic neuropathy cannot always be prevented. Previous studies have revealed that these two types of neuropathies are caused by completely different expression mechanisms. Therefore, in prevention and treatment research, it is necessary to take neuropathy-specific measures.

In addition, the observation of clinical neuropathy symptoms, particularly, evaluation of pain sensations using the von Frey test during animal experiments is inadequate. Clinical neuropathy symptoms include numbness-like sensations, which, unlike pain, are difficult to assess in current animal behavioral experiments. The gap between the basic and clinical aspects of these assessment methods may also influence the development of OIPN drugs. Therefore, it is necessary to improve behavioral experiments that can better reflect clinical symptoms.

Although research in this topic has become more active, there are still no promising drugs for CIPN, including OIPN, and further research is required to resolve this. Furthermore, new therapeutic agents such as immune checkpoint inhibitors for cancer treatment are being developed successively, and it is necessary to pay attention to adverse events of the nervous system related to these agents. Hence, research on neuropathy, which is an adverse event in the oncology field, is an important research theme in the future.

Finally, to establish an effective preventive method in the future, it is important to elucidate a more detailed mechanism, examine preventive and therapeutic agents considering the mechanism, and accumulate more reliable evidence to improve the strategies for OIPN management which are currently unresolved.

## Author Contributions

NE carried out the literature search and wrote this mini review.

## Funding

This work was supported by the Japan Society for the Promotion of Science (JSPS) KAKENHI grants, JP17K08953 and JP21K06670.

## Conflict of Interest

The author declares that the research was conducted in the absence of any commercial or financial relationships that could be construed as a potential conflict of interest.

## Publisher's Note

All claims expressed in this article are solely those of the authors and do not necessarily represent those of their affiliated organizations, or those of the publisher, the editors and the reviewers. Any product that may be evaluated in this article, or claim that may be made by its manufacturer, is not guaranteed or endorsed by the publisher.

## Abbreviations

CaMKII: Ca^2+^/calmodulin-dependent protein kinase II
CIPN: chemotherapy-induced peripheral neuropathy
DRG: dorsal root ganglion
GJG: goshajinkigan
MATE1: multidrug and toxic extrusion 1
MnSOD: manganese superoxide dismutase
NMDA: *N*-methyl-D-aspartate
NOS: nitric oxide synthase
NR2B: NMDA receptor subtype 2B
OCT: organic cation transporter
OCTN: organic cation transporter novel
OIPN: oxaliplatin-induced peripheral neuropathy
Pt (dach) Cl_2_, dichloro (1: 2-diaminocyclohexane) platinum
TRP: transient receptor potential
TRPA1: TRP ankyrin 1.
